# Chemical Communication between the Endophytic Fungus *Paraconiothyrium Variabile* and the Phytopathogen *Fusarium oxysporum*

**DOI:** 10.1371/journal.pone.0047313

**Published:** 2012-10-15

**Authors:** Audrey Combès, Idrissa Ndoye, Caroline Bance, Jérôme Bruzaud, Chakib Djediat, Joëlle Dupont, Bastien Nay, Soizic Prado

**Affiliations:** 1 UMR 7245, MCAM, MNHN CNRS, Muséum National d’Histoire Naturelle, Paris, France; 2 Plateforme de microscopie électronique, Muséum National d’Histoire Naturelle, Paris, France; 3 UMR 7205, OSEB, MNHN CNRS, Muséum National d’Histoire Naturelle, Paris, France; New York State Health Department and University at Albany, United States of America

## Abstract

*Paraconiothyrium variabile*, one of the specific endophytic fungi isolated from the host plant *Cephalotaxus harringtonia*, possesses the faculty to inhibit the growth of common phytopathogens, thus suggesting a role in its host protection. A strong antagonism between the endophyte *P. variabile* and *Fusarium oxysporum* was observed and studied using optic and electronic microscopies. A disorganization of the mycelium of *F. oxysporum* was thus noticed. Interestingly, the biological effect of the main secondary metabolites isolated from *P. variabile* against *F. oxysporum* did not account for this strong antagonism. However, a metabolomic approach of pure fungal strains and confrontation zones using the data analysis tool XCMS were analyzed and pointed out a competition-induced metabolite production by the endophyte in the presence of the phytopathogen. Subsequent MS/MS fragmentations permitted to identify one of the induced metabolites as 13-oxo-9,11-octadecadienoic acid and highlighted a negative modulation of the biosynthesis of beauvericin, one of the most potent mycotoxin of *F. oxysporum*, during the competition with the endophyte.

## Introduction

Endophytic fungi have been shown to establish mutualistic associations with their host plants, thus conferring fitness benefits, such as tolerance to biotic and abiotic stresses, nutrient acquisition and increased growth and yields. It is now clear that endophytes can protect hosts against fungal pathogens. For instance the presence of endophytes in the leaves of *Theobroma cacao* L. significantly decreases both leaf necrosis and leaf mortality when the plant is challenged by the oomycete phytopathogen *Phytophthora* sp [1]. Similarly, endophytic isolates confer resistance to pathogens in barley (*Hordeum vulgare* L.) [2]. There have been only few investigations on the physiological mechanisms of the endophyte host protection against phytopathogens. Although it has long been known that fungal secondary metabolites are crucial to the pathogenicity of many fungi, only few studies on the role of endophytic secondary metabolites in the endophyte-plant interaction have been reported. In many case, compounds isolated from endophytic fungi are fungicidal, herbicidal or antibacterial, thus supporting the idea that these metabolites play a role in the host plant defense [3], [4]. The fact that the significant antifungal activity of the maize endophyte *Acremonium zeae* W. Gams & R.D. Sumner against the phytopathogens *Aspergillus flavus* Link and *Fusarium verticillioides* (Sacc.) Nirenberg is mediated by the production of complex polyketides is a good illustration of this point [5].

Moreover, interactions among microbes, including endophytes, encompass antagonistic or competitive outcomes. Thus, interacting fungi may limit each other’s growth through antibiosis, in the course of which secondary metabolites limit the mycelial growth, spore production or spore germination of the opposing fungi. For example in wood, competitive mycelial interactions are very important in the overall development of fungal communities and it has been shown that secondary metabolites of some wood-decaying fungi can act as total inhibitors or stimulators of the growth of others fungi [6].

In this context and in order to study the mutualism between a plant host and its community of endophytic fungi, we initiated a program based on the cultivable fungal diversity of *Cephalotaxus harringtonia* (Siebold & Zucc.) Koidz, an Asian medicinal plant traditionally used against leukemia. Our choice of the *Cephalotaxus harringtonia* has mainly been guided by the chemical and phytochemical background of our team. Indeed, the phytochemistry of this tree is well-known by our chemist and few diterpenes and original alkaloids have already been isolated in our laboratory [7]–[9]. More than 640 isolates were isolated and identified with the ITS rDNA sequences, a species-level molecular marker of fungi [10]. Among them, *Paraconiothyrium variabile* Riccioni, Damm, Verkley & Crous appeared to be relatively specific to the plant *C. harringtonia*. Moreover, this endophytic fungus had also the ability to inhibit phytopathogens growth, suggesting a protective role for the plant. Indeed, a strong antagonism between *P. variabile* and *Fusarium oxysporum* Schltdl. was observed. We demonstrate in this report that this is not mediated by the endophyte main secondary metabolites we isolated, but by minor fungal metabolites specifically induced during the interaction with the phytopathogen. Metabolomic profiles of pure fungal cultures and confrontation zones were differentially analyzed by LC/MS and LC/MS-MS and led to demonstrate for the first time the role of an oxylipin in the modulation of the production of beauvericin, a well-known mycotoxin of *F. oxysporum*.

## Materials and Methods

### General Experimental Procedures

Optical rotations were determined with a Perkin–Elmer Model 341 Polarimeter and the [α]_D_ values are given in deg.cm^2^.g^−1^. Mass spectra were recorded on an API Q-STAR PULSAR i of Applied Biosystem. For the CID spectra, the collision energy was 40 eV and the collision gas was nitrogen. The ^13^C NMR spectra were recorded on a Bruker AC 300 spectrometer operating at 75.47 MHz (for ^13^C). The ^1^H and 2D-NMR spectra were recorded at 298 K on a Bruker AVANCE 400 spectrometer operating at 400.13 MHz. For HMBC experiments the delay (1/2 J) was 70 ms and for NOESY experiments the mixing time was 150 ms.

### Fungal Material

The fungus was isolated in October 2008 as an endophyte from a needle of *Cephalotaxus harringtonia* var. *drupacea* referenced under N° 2686 in the Arboretum de Chèvreloup (MNHN) [10]. Both morphological assessment and internal transcribed species (ITS) sequencing were performed to characterize the isolate as *Paraconiothryrium variabile.* It is now maintained at the LCP culture collection (culture collection of the Muséum National d’Histoire Naturelle, Paris) under the number LCP 5644.

### Strains

The phytopathogen strains *Fusarium oxysporum* (LCP 531), *Fusarium moniliforme* J. Sheld. (LCP 3184), *Sphaeropsis sapinea* (Fr.) Dyko & B. Sutton (LCP 3819) came from the fungal culture collection of the Muséum National d’Histoire Naturelle, Paris. *Alternaria alternata* (Fr.) Keissl. (5D2C), isolated as an endophyte from *C. harringtonia* (tree n° 2685 in the Arboretum de Chèvreloup), is although known as a pathogenic fungus on various plants.

### Host Specificity

Extracts containing 10% wt/vol suspensions of mature leaves from the host plant *C. harringtonia* or from *Thuja plicata* D.Don ex Lamb. were incorporated into water agar before autoclaving (final concentration 10% vol/vol). Plugs of hyphae and agar (5/5 mm) were cultivated on media and colony diameters were compared to control (agar alone) after 12 days.

### Antagonism on Petri dish

60 mL of sterile PDA were poured into Petri dishes 120×120×17 mm (square). Spores of each strain were recovered from a piece of agar 5 mm sides in 1 mL of glycerol 20% and 10 µL of each suspension were inoculated on the agar at 25 mm from the edge. For co-culture on Petri dishes, the two strains were compared and placed at 25 mm of two opposite extremities. Since the endophyte grows more slowly, the phytopathogens were inoculated on agar 7 days after *P. variabile*. The dishes were then placed at 27°C and observed daily. The diameter of each colony were measured daily from the point of deposit and compared with strains from the co-culture. The same protocol was applied in divided Petri dish (9 cm) with two compartments containing the PDA medium.

### Optic Microscopy

The structure of the mycelium of *F. oxysporum* (cultured on agar) was observed with a binocular microscope Nikon Eclipse TE 300. This was then compared to that of mycelia obtained from dual culture with the endophyte. The images were captured with a CCD sensor (Photometrics CoolSnap HQ 1392×1040 pixels).

### Electronic Transmission Microscopy

From single and dual culture on agar at day fourteen, small plugs of mycelium of *Fusarium oxysporum* were cut and fixed with a mixture of glutaraldehyde 2.5%, picric acid 0.5%, and sucrose (0.18 M) in 0.1 M pH 7.4 Sörensen buffer. The fixation was followed by a postfixation with osmium tetroxide (1%). In both cases, samples were washed three times in Sörensen phosphate buffer (0.1 M, pH 7.4) in three 10 minutes-long successive baths and were dehydrated in ethanol by three baths (50°, 70°, 90° and 100°) and then embedded in an epoxy mixture (Spurr’s resin). Medium and ultrathin sections were sliced with diamond knives (Diatome) on a Reichert-Jung Ultracut microtome. The 0.5 microns semi-thin sections were stained with toluidine blue 1% and sodium borate (1%) in ethanol (70%) or with methylene blue-basic fuchsin staining. The 500–700 Angström ultrathin sections were stained with a saturated solution of uranyle acetate in 50% alcohol and then observed with a transmission electronic microscope (Hitachi H-7100). The pictures were taken using a Hamamatsu CCD camera.

### Cultivation of *P. variabile* and Isolation of Metabolites

The fungus *P. variable* was maintained in potato dextrose agar at 25°C. The agar was cut into small plugs and inoculated into 15 Erlenmeyer flasks (750 mL) containing modified Czapek Dox medium (D-glucose (40 g), L-alanine (2 g), KH_2_PO_4_ (1 g), MgSO_4_ (0.5 g), KCl (0.5 g), ZnSO_4_.7H_2_O (0.01 g), CuSO_4_.5H_2_O (0.05 g), thiamine (100 µg), biotine (10 µg) for 1 L of deionized water). After incubation at 25°C for 40 days on rotary shaker (160 rpm), the culture was centrifuged (7000 rpm, 20 minutes) to separate the mycelium and the filtrate. The culture filtrate was then extracted with ethyl acetate (3 × 1 L) and the combined organic extracts were dried over MgSO_4_ and evaporated, giving 4 g of crude extract.

This crude extract was subjected to a first chromatography on Sephadex LH20 (methanol) and afforded 8 fractions. Fractions 6 to 8 were assembled from their metabolic profiles on thin layer chromatography and subjected to chromatography on silica gel (dichloromethane/methanol 98/2) affording (**1**, 20 mg), (**2**, 2 mg), (**3**, 3 mg), (**5**, 5 mg). The subfraction 5 was further purified by column chromatography, leading to compounds (**4**, 9 mg) and (**6**, 2 mg).

### Inhibition of *F. oxysporum* Growth in 96 well Plates

A suspension of spores (obtained as above) were counted on a Malassez cell and adjusted to 10^4^ spores/mL. 100 µL of the suspension were spread over a 96-well plate. 4 µL of the metabolite of interest at 10 mg/mL in a DMSO solution were added in the first column and diluted by serial dilutions. Econazole was used as a positive control and DMSO as a negative control. The 96-well plates were placed at 27°C. Reading was done using a microscope (Hund Wetzlar, magnification×40). The MIC (minimum inhibitory concentration) was considered to be the concentration at which no mycelium was observed. Manipulations were performed in triplicate. Different combinations mixtures of compounds were also tested on the growth of *F. oxysporum* (4 µL, 10 mg/mL, 1∶1 v:v), notably all the six compounds together: **1**+**2**+**3**+**4**+**5**+**6**. The main compound of the tetralone family **1** was tested with all the others compounds: **1**+**2** to **1**+**6** but also mixed with the compounds of the different chemical series: **1+4+5+6**, **1+4+5**, **1+5+6**. Moreover, the **4**+**5+6**, **4**+**5**, **4**+**6** and **5+6** combinations were also tested. Commercial 13-oxo-ODE (BertinPharma) was also evaluated against *F. oxysporum* using the same protocol.

### Extraction of Metabolites during the Microbial Competition on Dual Culture

From dual and single cultures five plugs of agar and mycelium (5/5 mm) of *P. variabile* and *F. oxysporum* were cut and extracted with ethyl acetate (3×50 mL) under sonication. After filtration, the solvent was removed by evaporation under vacuum and the crude extract was diluted in methanol at a concentration of 5 mg/mL before analysis by LC/MS or LC/MS-MS.

### Effect of the 13-oxo-ODE Oxylipin on the Beauvericin Production

A suspension of spores (obtained as above) were counted on a Malassez cell and adjusted to 10^6^ spores/mL. 5 mL of the suspension were spread over a square Petri dish of PDA (60 mL). 10 µL of commercially 13-oxo-ODE (BertinPharma) at 0.01 mg/mL in methanol were then added onto the solid media plates immediately or ten days after. On the thirteenth day, plug of agar and mycelium (10/10 mm), of the specific region to which the 13-oxo-ODE was added, were cut extracted and concentrated as described above. The crude extract then was diluted in methanol at a concentration of 10 mg/mL before analysis by LC/MS.

### LC/MS and LC/MS/MS Experiments on Metabolite Extracts Expressed during the Microbial Competition

Crudes extracts were analyzed on a liquid chromatography (UltiMate 3000®, Dionex, Germany) coupled to a Quadrupole-Time of flight (Q-TOF) hybrid mass spectrometer (pulsar i, Applied Biosystems, France) equipped with an electrospray ionization source (ESI). The volume injected was 5 µL. Extract separation was conducted on an ACE3-C18 column, 3 µm × 5 cm × 0.5 mm (ACE, Scotland). Mobile phases were milliQ water containing 0.1% (v/v) formic acid (A) and acetonitrile containing 0.07% (v/v) formic acid (B). The separation was achieved at a flow rate of 40 µL.min^−1^ with the following gradient: 10% B during 5 minutes, 10 to 50% B in 20 minutes, 50 to 75% B in 20 minutes, 75% B during 5 minutes, 75 to 10% B in 5 minutes and 10% B during 10 minutes. LC/MS and LC/MS-MS analyses were carried out in positive electrospray ionisation mode. For LC/MS analyses, the capillary voltage was set to 2500 V, the ion spray potential and declustering potential were 5200 V and 40 V. Full scan mass spectra were performed from 50 to 1300 *m/z* at 1 s/scan in continuum mode. For LC/MS-MS analyses cone voltages of 42 V and collision energies of 40 eV were applied. LC/MS/MS fragmentations were also made on commercially 13-oxo-ODE (BertinPharma) and beauvericin (BertinPharma) standards.

### LC/MS Data Processing and Analysis

Analyst QS® software (ABsciex) was used to convert the original mass spectrometry data files (*.wiff) to a more exchangeable format (*.cdf) with the included File translator utility. The data were then processed by the open-source XCMS software [11] (XCMS parameters for the R language were implemented in an automation script). Without using internal standards, the method dynamically identified hundreds of endogenous metabolites for use as standards, calculating a nonlinear retention time correction profile for each sample. This tool allowed us to extract ions statistically over or under-represented between two groups of samples (the software calculates *t*-test and *p*-value). The final result data table was then imported to Excel®. The analysis was performed on LC/MS data issued from two independent culture experiments.

In order to quantify the beauvericin in our sample, the “LC/MS reconstruct” tool in BioAnalyst 1.1® was applied to the LC/MS data. This algorithm interprets the data to enumerate the molecular species represented and the total intensity (counts) integrated over the *m/z* range of the ions to contribute to the molecular mass information. Beauvericin was quantified using beauvericin as external standard. Response between the injected quantity (ng) and total intensity (counts) was calculated for each experiment and was linear within the range of 2 ng to 100 ng (y = 1.88.10^−4^×–1.31, r^2^ = 0.99). Two replicates were performed for each injected quantity.

### LC/MS-MS Data Processing and Analysis

The exact mass obtained by MS analysis and the couple formed by relative intensity and mass of each ions issued from MS-MS fragmentation were submitted to the metabolite search tool of METLIN (http://metlin.scripps.edu/metabo_advanced.php) [12]. The tolerance on mass of ions resulting from the MS-MS fragmentation and on the mass of precursor ion was respectively set to 0.05 Da and to 10 ppm.

## Results

### Host-preference of *Paraconiothyrium Variabile*

To assess the host preference of endophytes, we compared the endophyte communities of *C. harringtonia* with those of neighbouring conifers trees in the arboretum (*Pinus apulcensis* Lindl., *Thuja plicata*, *Taxus baccata* L.) [10]. Accordingly, *P. variabile* was selected for further studies because it was present in *C. harringtonia* and absent from the leaves of the neighbouring conifers. Moreover, as described by Arnold et al. [1], we used an *in vitro* experiment to investigate whether the endophyte growth was sensitive to the leaf content of the host plant. Thus, the growth of *P. variabile* isolate was monitored on media containing leaf extracts of *C. harringtonia*, *Thuja plicata* or on agar alone. The colony diameter was measured after nine days. A quicker growth of *P. variabile* was observed in the presence of *C. harringtonia* leaf extract (colony diameter: 36.6±1.52 mm,) in comparison with the controls (colony diameter with *Thuja plicata* leaf extract: 32.3±2.08 mm, p<0.05, and on agar alone: 32.0±1.0 mm, p<0.01).

### Antagonism between the Endophytic Fungus *P. variabile* and Phytopathogens

**Figure 1:**
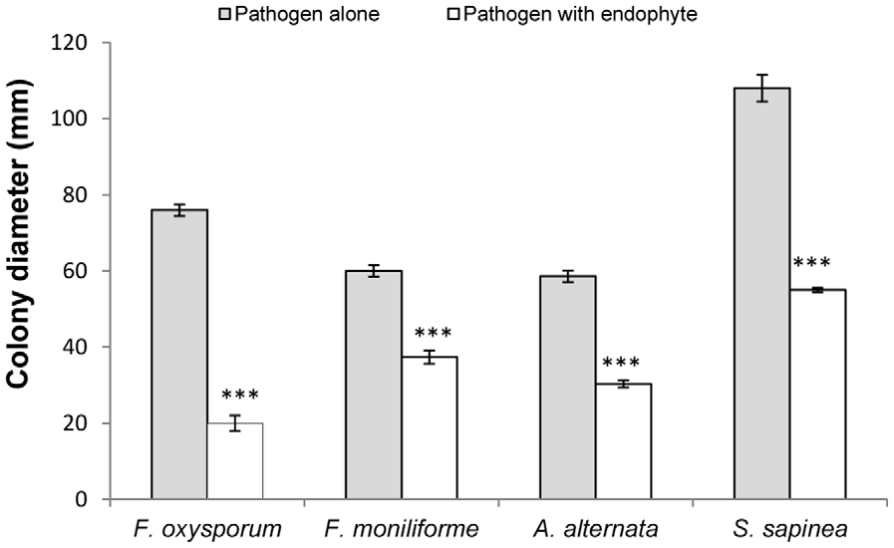
Antagonism observed in dual culture of *P. variabile* with different strains of phytopathogens. Colonies diameters of different phytopathogens strains were measured at day fourteen in absence (gray) or in presence (white) of the endophyte *P. variabile*. Error bars indicate standard deviation (n = 3). Asterisks indicate a statistically significant decrease of the growth of the phytopathogen in the presence of the endophyte by t-test (***p<0.001).

**Figure 2:**
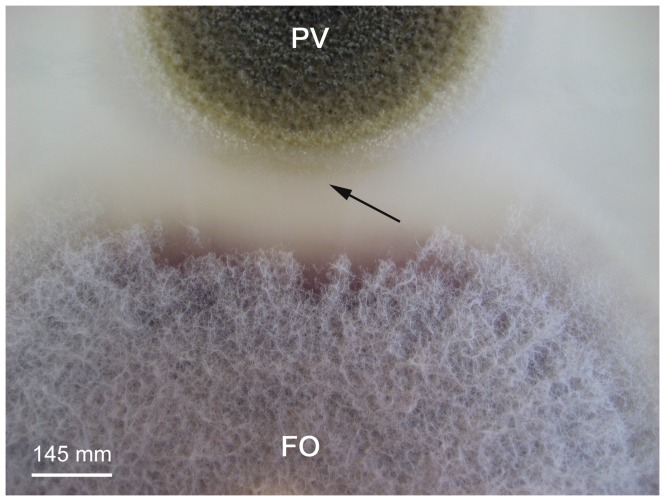
Antagonism between the endophytic fungus and the phytopathogen *F. oxysporum* in dual culture. *P. variabile* (PV) and *F.oxysporum* (FO). The two strains were placed at 25 mm of two opposite extremities of a Petri Dish on PDA medium and were observed at day fourteen. Disorganization of the phytopathogen’s mycelium structure can be observed. Arrow represents a pigmented line which could correspond to the biosynthesis of diffusible endophytic metabolites.

**Figure 3:**
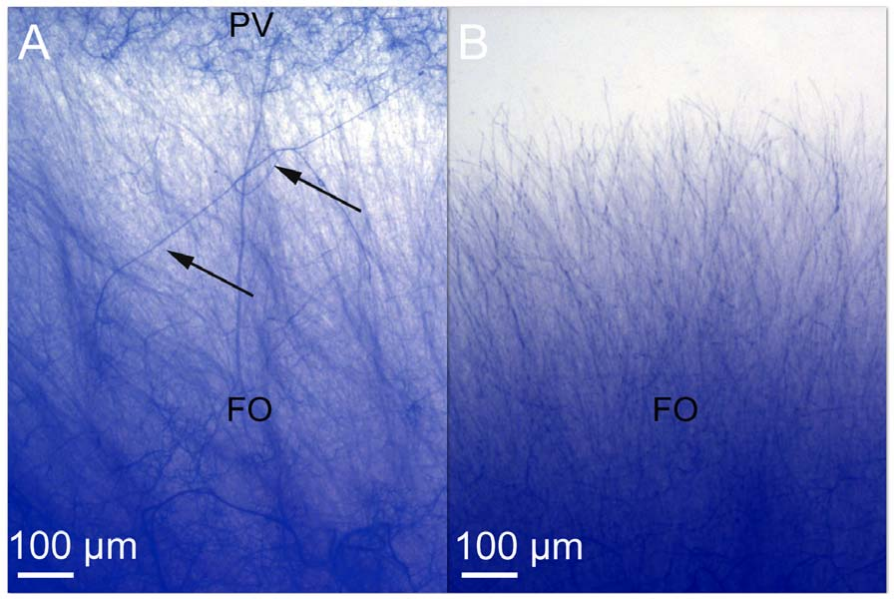
Observation by optic microscopy of the mycelium of *F. oxysporum* in dual culture or single culture. (A) *F. oxysporum* hyphae from dual culture with the endophyte. (B) *F. oxysporum* hyphae control. (PV) *P. variabile* and (FO) *F.oxysporum*. Optic micrographs of the mycelium of *F. oxysporum* from dual culture or single on agar plate with the endophyte *P. variabile* (14 days, 27°C). Arrows show disorganization of the hyphae in the presence of the endophyte.

**Figure 4:**
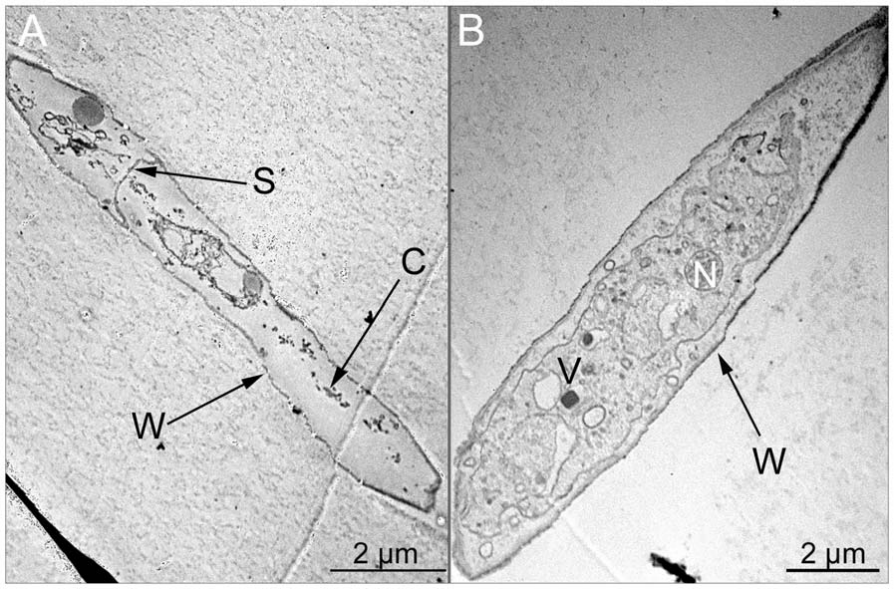
Transmission electron micrographs of the hyphae of *F. oxysporum* from dual or single culture. (A) *F. oxysporum* mycelium from dual culture with the endophyte. (B) *F. oxysporum* mycelium control. Transmission electron micrographs of the mycelium of *F. oxysporum* from dual or single culture on agar plate with the endophyte *P. variabile* (14 days, 27°C) and showing cell death in the presence of the endophyte. N: nucleus. W: wall. S: septum. C: cellular fragments. V: vesicule.

The endophyte *P. variabile* was assayed for its capacity to inhibit the growth of common phytopathogens such as *Fusarium oxysporum*, *Fusarium moniliforme*, *Sphaeropsis sapinea* and *Alternaria alternata* in dual cultures on Petri dishes. In all cases, the growth of the phytopathogens was inhibited as shown in Figure 1. Indeed, the colony growth of the phytopathogens was clearly diminished at fourteen days and it was not recovered even after sixty days (data not show). A pigmented line, which could correspond to the biosynthesis of diffusible endophytic metabolites, was also observed (Figure 2). The most potent antagonism effect of *P. variabile* was observed on *F. oxysporum* growth which displayed disorganization of its mycelium structure in the presence of the endophyte. The effect of the endophyte *P. variabile* on the mycelium of *F. oxysporum* was confirmed by microcopy. As depicted in Figure 3, a disruption of the mycelium of *F. oxysporum* and an organization in “strands” of the hyphae of *F. oxysporum* were observed in the presence of *P. variabile* (Fig. 3A). In order to further study the antagonistic effect of *P. variabile* on *F. oxysporum*, we used transmission electron microscopy on the hyphae of the phytopathogen from single or dual cultures. This revealed a population of dead hyphae devoid of cytoplasm and organelle in the sections of mycelium obtained from the co-cultures (Fig. 4A). Conversely micrographs from *F. oxysporum* alone displayed classical mycelium organization of a filamentous fungus. (Fig. 4 B).

### Isolation of the Metabolites of *P. variabile*

**Figure 5:**
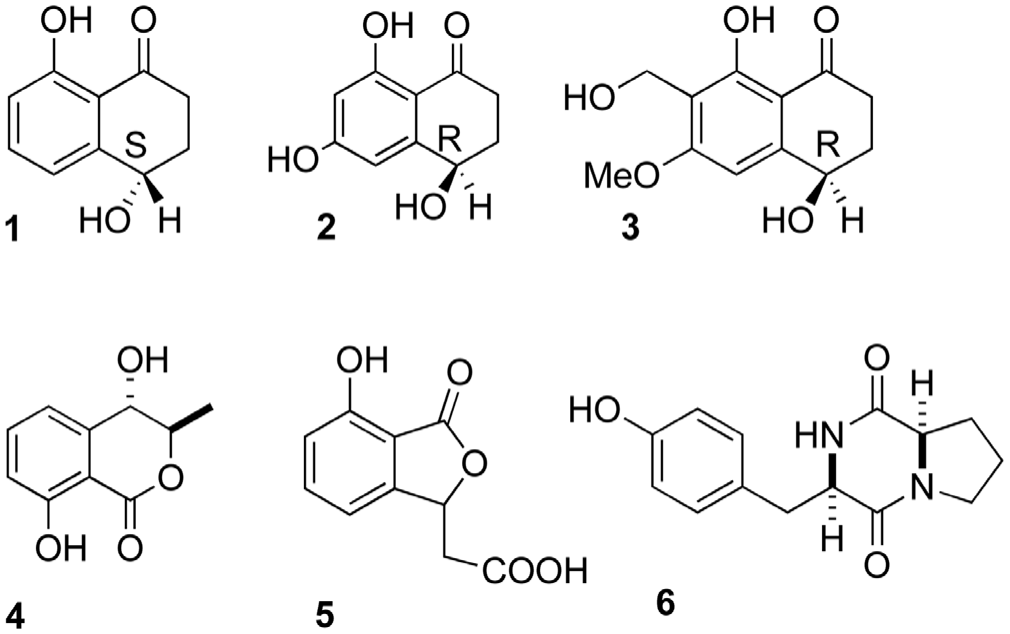
Structures of 1–6 isolated from *P. variabile.* All structures were assigned by detailed analysis (NMR spectroscopic and mass data).

Cultures of *P. variabile* were extracted with ethyl acetate. Successive chromatographies of the crude extract on silica gel and Sephadex LH-20 yielded the following six compounds: isosclerone (**1**), 6-hydroxyregiolone (**2**), norparvulenone (**3**), 4-hydroxymellein (**4**), isoochracinic acid (**5**) and *cyclo*(Tyr-Pro) (**6**) (Figure 5). The structures of these compounds were determined by mass spectrometry and nuclear magnetic resonance (NMR). The absolute configuration of compounds **1–3** was determined by circular dichroïsm (CD) on the basis of comparison with the CD spectrum of isosclerone (**1**) [13] and on the value of the optical rotation. CD spectra of **1** showed strong positive Cotton effect at 213 nm and low Cotton effect at 258 nm indicating a *S* configuration on C-4 (Figure S1). This is in agreement with the experimental value of optical rotation [α]^22^_D_: +18.5 (c 3.25, CHCl_3_). In return, the CD spectrum of norparvulenone (**3**) presented a perfect image mirror of (4*S*)-isosclerone indicating an *R* configuration at C-4. Moreover, the value of the optical rotation of (**2**) [α]^22^_D_: +11 (c 0.03, MeOH) also suggests an *R* configuration according to literature precedent on regiolone/isosclerone [13].

### Activity of the Isolated Metabolites against *F. oxysporum*

The growth inhibition of *F. oxysporum* by the main endophytic secondary metabolites was evaluated on 96 wells plates. At the highest concentration tested (200 µg/mL) no inhibition was observed for compounds **1**–**6**, in comparison with the control (econazole: 3 µg/mL). A synergistic allelopathic activity for all these metabolites was also evaluated by assaying the effect of the mixture of compounds on the growth of the phytopathogen and notably the effect of all 6 compounds together. Again, no significant effect was observed. The potential of *P. variabile* to produce antifungal metabolites as volatile organic compounds (VOCs) was then evaluated. Thus, bioassays on divided PDA Petri dishes inoculated with *P. variabile* in one component and *F. oxysporum* in the other were performed. No growth difference of the phytopathogen was observed in dual divided cultures (colony growth of *F. oxysporum* at ten days: 37.30 mm±0.57) or in culture alone (colony growth of *F. oxysporum* at ten days: 37.30 mm±0.55), thus suggesting that the inhibition observed was mediated by diffusible metabolites only and not by VOCs.

### Activity of the 13-oxo-ODE against *F. oxysporum*

The commercial 13-oxo-ODE was also tested against the growth of the phytopathogen *F. oxysporum* on 96 wells plates. At the highest concentration tested (200 µg/mL) no inhibition was observed for compounds 13-oxo-ODE, in comparison with the control (econazole: 3 µg/mL).

### Analysis of Metabolites Induced during the Competition

**Figure 6:**
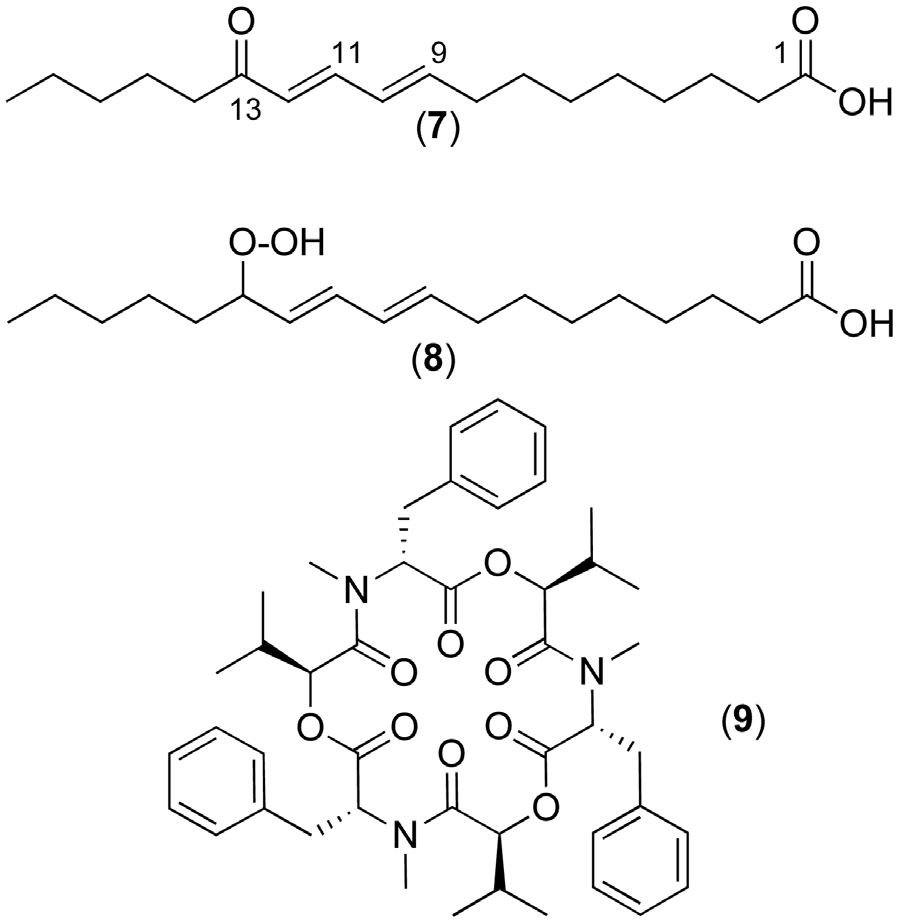
Compounds implied in the chemical communication between the endophyte *P. variabile* and the phytopathogen *F. oxysporum.*

In an attempt to demonstrate that metabolites were specifically induced during the interaction, we compared the LC/MS profiles of the metabolites extracted from the culture of pure fungal strains and from the confrontation zones using XCMS [11]. This tool corrects the retention time between samples and allows direct comparison of relative metabolite ion intensities in order to identify variations in specific endogenous metabolites. Indeed, comparison of ions generated from metabolites of *F. oxysporum* and *P. variabile* in competition showed that only 25 ions were specifically found in the crude extract of *F. oxysporum*, 99 ions were present only in the crude extract of *P. variabile* and 47 others ions were common to both crude extract. In this study we focused on secondary metabolites induced by *P. variabile* during the competition and which could be involved in the antagonism against *F. oxysporum*. Therefore the ions issued from *F. oxysporum* were excluded of the analysis. Under these conditions 99 ions were induced by *P. variabile* during the competition. Manual analysis of these ions led us to eliminate 57 ions related to hydrophilic molecules which were not retained on the column (retention time <2 minutes), to remove duplicates (molecules present in different charge and/or isotopic state in LC/MS data; 2 ions) and to eliminate 22 ions with a low intensity (maximum intensity <200 counts and ratio Signal/Noise <5). The mass spectra corresponding to the remaining 18 ions were then studied, allowing for determining the charge state of the ions detected, grouping ions corresponding to identical molecules (18 ions for 12 molecules) and deducing the molecular weight of potentially interesting metabolites (Table 1). Remarkably, 12 metabolites were specifically induced by the endophytic fungus in dual cultures suggesting that part or all of them could be responsible for the strong antagonism observed. These 12 metabolites were fragmented in MS/MS mode and their monoisotopic mass and MS/MS fragments were submitted to the METLIN Metabolite database. Through this database search, the peak with *m*/*z* of 295.2257 was identified as 13-oxo-9,11-octadecadienoic acid (13-oxo-ODE, **7**, Figure 6) with a score of 100 and an error of Δ3 ppm on the exact mass. According to this result, the LC/MS-MS analysis of the commercially available 13-oxo-ODE, was also performed and compared to our sample. The exact mass (*m/z* = 295.2277) and the retention time (rT = 32.4 min) of this molecule was consistent with those of our observed compound (*m/z* = 295.2257, rT = 32.9 min). In the fragmentation spectra of 13-oxo-ODE, the characteristic ion at *m/z* 179.12 corresponding to the cleavage of the C13–C14 bond followed by decarboxylation was found. In the MS-MS spectrum of our compound and in the 13-oxo-ODE, successive fragmentation of the ion 179.12 led to ions 151.10, 109.09, 95.07 and 81.06 corresponding to successive cleavage on the carbon chain (Table 2). This mechanism of fragmentation based on the preferential rupture of the C13–C14 bond was consistent with those previously described for this molecule [14], [15]. Moreover, in a same way, compound at *m/z* 312.3350 could correspond to 13-hydroperoxy-9,11-octadecadienoic acid (**8**, Figure 6), a well-known intermediate in the biosynthesis of the 13-oxo-ODE oxylipin (**7**) [16].

**Table 1:** Metabolites induced by *P. variabile* during the competition with the phytopathogen *F. oxysporum.*

rT (min)	*m*/*z* median for ions on mass spectra	Molecular mass of the corresponding metabolite	Identification
38.5	277.2161	276.3912	nd
32.9	295.2257	294.3957	13-oxo-9,11-octadecadienoic acid (Δppm = 3)
	296.2316		
51.3	312.2904	310.4735	nd
42.0	313.2389	312.3350	13-hydroperoxy-9,11-octadecadienoic acid (Δppm = 4)
	314.2589		
45.3	358.3382	357.5500	nd
	359.3270		
56.7	377.3199	376.6255	nd
	378.3227		
56.6	393.3169	392.4076	nd
49.9	396.3339	394,4600	nd
48.5	411.3261	410.5850	nd
38.8	625.4610	624.7521	nd
32.6	657.3982	656.8960	nd
	658.4256		

nd: non identified.

**Table 2:** Fragmentations of the positive ion (*m*/*z* = 295.22) identified as 13-oxo-9,11-octadecadienoic acid and of the commercial standard.

Fragments	ion *m/z* = 295.22	13-oxo 9,11-octadecadienoic acid
[M + H] +	295.22	295.22
	279.17	279.17
[M − CH_3_(CH_2_)_4_ − COOH +H] ^+^		179.12
[M − CH_3_(CH_2_)_4_ − (CH_2_)_ 2_COOH +H]^+^	151.08	151.10
[M − CH_3_ − CHCH(CH_2_)_7_COOH +H]^+^	111.03	111.07
[M − CH_3_ (CH_2_)_4_ − (CH_2_)_5_COOH +H]^+^	109.09	109.09
[M − CH_3_CH_2_−CHCH(CH_2_)_7_COOH +H]^+^	97.09	97.05
[M − CH_3_(CH_2_)_4_ − (CH_2_)_6_COOH +H]^ +^	95.08	95.07
[M − CH_3_ − CHCHCHCH(CH_2_)_7_COOH +H]^+^	85.09	85.05
[(CH_2_)_2_ COCHCH + H]^+^	83.04	83.04
[M − CH_3_(CH_2_)_4_ − (CH_2_)_7_COOH +H]^+^	81.06	81.06
[CH_3_(CH_2_)_4_ +H ]^+^		71.08
[(CH_2_)_2_CO +H]^+^	57.07	57.06
[(CH_2_)_2_CHCH +H]^+^	55.05	55.05

### Modulation of Mycotoxin Biosynthesis in the Course of the Interaction between the Phytopathogen *F. oxysporum* and the Endophyte *P. variabile*

LC/MS profile of *F. oxysporum* was screened for the presence of known mycotoxins by MS comparison with those described in the literature. Beauvericin, a common hexadepsipeptide mycotoxin produced by *Fusarium* spp, appeared as the main mycotoxin produced by *F. oxysporum*. MS/MS fragmentations of the positive ion (*m/z*: 784.41, rT = 39.3 min) led to ion fragments formed successively by the loss of CO (*m/z*: 756) and by the cleavage of the amide and esters bonds (*m/z*: 623, 523, 362, 262) (Figure S2). This pattern of fragmentation is consistent with the one previously described for beauvericin [17] and allowed us to confirm the nature of this molecule. The concentration of beauvericin produced by *F. oxysporum* alone was quantified by LC/MS and was determined as 2.37±0.47 µg/mg of dry weight. This concentration strongly decreased to 0.15±0.02 µg/mg in dry weight when *F. oxysporum* was in competition with *P. variabile*.

### Modulation of Mycotoxin Biosynthesis by the 13-oxo-ODE

The concentration of beauvericin produced by *F. oxysporum* alone was also quantified by LC/MS and was determined as 32.53±3.29 µg/mg of dry weight. This concentration decreased to 14.53±1.46 µg/mg of dry weight when *F. oxysporum* was in presence of 50 ng of 13-oxo-ODE.

Moreover, the same decrease of the production of beauvericin was observed when the 13-oxo-ODE was added ten days after fungal inoculation. In this case concentration of beauvericin produced by *F. oxysporum* alone was determined as 36.86 µg/mg ±3.72 µg/mg of dry weight and as 20.60 µg/mg ±2.08 µg/mg of dry weight in the presence of 50 ng of the 13-oxo-ODE.

## Discussion

The role of fungal metabolites in the symbiotic relationship of endophytes with the host plant remains yet under-investigated. In this context, we have studied the role of the host specific endophyte *P. variabile* on the plant protection. Indeed, this endophyte was mainly found in *C. harringtonia* and not in the neighboring trees of the site and seemed to be sensitive to the leaf content of the host plant. To investigate a possible protective role of *P. variabile* for the plant, *in vitro* co-cultures with phytopathogens were used to detect the production of antagonistic metabolites. Indeed, the endophyte was able to inhibit the growth of several phytopathogens frequently found in conifers. The strongest antagonism was observed with *F. oxysporum*, which suffered important perturbations of the mycelium structure.

To assess the possible role of the endophyte secondary metabolites, a chemical study was undertaken on the main compounds. Although *P. variabile* had not been studied before, this work followed reports on the chemical characterization of other *Paraconiothyrium* metabolites which mainly yielded terpenoids [18], [19]. However recently *P. variabile* was shown to produce paclitaxel [20]. Interestingly, six known compounds belonging to four different chemical classes were isolated. The tetralone derivatives (**1**, **2**) are common fungal metabolites isolated from a wide variety of fungi [21]–[25]. Absolute configuration of the chiral center was assigned as *S* for **1** and **2** but *R* for the rarer norparvulenone (**3**). 5-Methylmellein (**4**), benzofurane (**5**) and diketopiperazine (**6**) were also isolated from other fungi [26]–[28].

All these compounds were evaluated for their ability to inhibit the growth of *F. oxysporum* but none of them were effective at concentrations as high as 200 µg/mL.

Moreover, since chemically mediated interactions between microorganisms are characterized by the release of allelopathic compounds, an effect is rarely observed with a single compound and can result from synergy [29]. Accordingly, the possible synergistic allelopathic activity of these compounds was tested but again, no antifungal property was observed.

On the other hand, some endophytic microbes have shown the ability to produce volatile organic compounds (VOCs) which can be antimicrobial, especially against phytopathogens [30], [31]. Therefore, we checked for the production of VOCs using a two compartments Petri dish containing PDA and inoculated with *P. variabile* in one part and *F. oxysporum* in the other. No effect on the growth of *F. oxysporum* was observed in the presence of the endophyte in the other compartment suggesting that the antagonism is indeed mediated by diffusible metabolites rather than by VOCs. It was suggested that the secondary metabolites responsible for antagonistic effects may only be produced in response to interactions with other stressing microorganisms. The growth of different microorganisms together forces direct interactions that may induce the productions of compounds not previously observed when the strains are grown independently [32]. Indeed, several co-culturing experiments have been reported, resulting in an increase of metabolite production, including new compounds [33]–[35]. Moreover, recent works have demonstrated that some biosynthetic pathways (in particular PKS and NRPS) are silent when the fungi are cultured under standard laboratory conditions. The corresponding genes would only be expressed upon stimuli such as environmental stresses or biotic signals [36]. Other example attests of the up-regulation of the endophyte secondary metabolism pathway during growth *in planta* [37]. In addition, a recent study highlighted the variation of metabolite expression detected by LC/MS during the interaction between the phytopathogen *Fusarium verticillioides* and the endophyte *Ustilago maydis* (DC.) Corda [38]. Thus, the complexity of the crude extract and the inactivity of the main purified compounds of *P. variabile* against the phytopathogen led us to study the metabolites specifically induced *in situ* during interaction between both fungi. We used a metabolomic profiling approach using the data tool XCMS to highlight them. It should be noted, that a similar approach was successfully developed by Glauser et *al.* for the chemical investigation of zone lines formed by the confrontation of fungi [39]. Thus, we put in evidence the metabolites produced by the endophytic fungus *P. variabile* during the interaction with *F. oxysporum* and compared them to those produced by the fungus in single culture. Remarkably, 12 metabolites were specifically induced by the endophytic fungus in dual cultures suggesting that part or all of them could be responsible for the strong antagonism observed (Table 1). Two of them could be identified by LC/MS and LC/MS/MS as 13-oxo-9,11-octadecadienoic acid (**7**) and 13-hydroperoxy-9,11-octadecadienoic acid (**8**) (Figure 6). These compounds belong to the oxylipin family which is originated by the oxidative metabolism of polyunsaturated fatty acids and are well known for their role in regulating development processes as well as in environmental responses in mammals and plants [16]. This family of compounds is supposed to be synthetized *de novo* from oleic and polyunsaturated fatty acids upon activation by external or internal stimuli, which is in accordance with our results [40].

Evaluation of the antifungal activity of the commercial sample of 13-oxo-9,11-octadecadienoic acid against *F. oxysporum* pointed out the absence of growth inhibition of the phytopathogen at the highest concentration tested (200 µg/mL, 0.7 mM). This result is consistent with those previously described by Prost et al. [41] and therefore suggest that the antagonism observed and the cell death of the hyphae of *F. oxysporum* in co-culture were mediated by others induced metabolites than the oxylipins.

Reports on the fungal oxylipins are still scarce especially concerning their physiological function as compared with the situation in plants and mammals, except for their role in *Aspergillus nidulans* (Eidam) G. Winter whose production of oxylipin mixtures (psi factor) is known to regulate both sexual and asexual spore development [40].

However, recent reports have further supported a signaling role for fungal oxylipins in the regulation of secondary metabolism cascades. For example, in *A. nidulans*, mutations of Ppo (for psi producing oxygenase) led to strong reduction in the production of the mycotoxin sterigmatocystin (ST). Analysis of transcripts in the mutant showed decreased expression levels of the ST biosynthetic gene [42] suggesting that the oxylipin species regulate secondary metabolites at the transcriptional level. Nevertheless, the effect of oxylipins on the host-pathogen interaction has been little studied excepted *in planta* in which, for example, linoleic acid and its 9- and 13-hydroperoxy derivatives from plant are able to regulate transcriptionally the production of ST and aflatoxin (AF) biosynthesis in *Aspergillus* [16]. As fungal enzymes and their corresponding oxylipins are highly similar to those produced by plant species and as oxylipins are able to control the pathogenesis of phytopathogens, we speculated that the oxylipin 13-oxo-ODE produced during the interaction can affect the production of mycotoxins from *F. oxysporum*. In this context, we explored the modulation of mycotoxin production by the phytopathogen alone or in the course of the competition with the endophyte *P. variabile*. First, we screened the LC/MS profile of *F. oxysporum* for the presence of mycotoxins by mass comparison with literature data. Beauvericin (**9**), a cyclodepsipeptide mycotoxin produced by *Fusarium* spp and belonging to the enniatin family, was found and its structure was validated by MS/MS fragmentations (Figure S2). Moreover, we have put in evidence a strong decrease of the concentration of beauvericin (90%) produced by the phytopathogen during the competition with the endophyte as compared to the concentration in *F. oxysporum* alone. This observation allowed us to hypothesize that just as in plants, oxylipins overexpressed by the endophytic fungus *P. variabile* could induce a decrease in the production of mycotoxins by *F. oxysporum*.

The biological role of mycotoxin still remains elusive and the most commonly held hypothesis is that mycotoxin-producing fungi are better protected against organism sharing the same trophic niche [43]. Nevertheless, a recent “oxidative stress theory of mycotoxin biosynthesis” has emerged and point out the pivotal role of oxidative stress. Among others things, this theory suggests that in response to oxidative stress (such as oxylipin production from unsaturated fatty acids) a differentiation process in fungi is promoted, leading to changes in morphogenesis and production of secondary metabolites (i.e. mycotoxins) [44].

Nevertheless to validate this down regulation of the mycotoxin, we quantified the beauvericin produced by *F. oxysporum* by LC/MS in the presence of 50 ng of 13-oxo-ODE. This concentration was selected from the quantification of this oxylipine produced during the microbial competition between the endophyte and the phytopathogen (data not shown). Under these conditions a decreased of the production of beauvericin was observed if the oxylipine was added immediately or ten days after of the growth of the fungus. In the two cases beauvericin production is significantly diminish (respectively 45% and 55%) in the presence of 13-oxo-ODE. In this context, and from our results, we can suggest that the down-regulation of mycotoxin production by endophytic oxylipin signal provides an ecological advantage for endophytic fungus in the microbial interaction with the phytopathogen. However a stronger 90% inhibition of beauvericin production was measured in the course of the antagonism between *P. variabile* and *F. oxysporum*, suggesting that additional mechanisms could be involved for which additional investigations are in progress.

### Conclusion

In order to better understand the mutualism between a plant host and its community of endophytic fungi we initiated a campaign of isolation and characterization of the endophytes of *C. harringtonia*. From the experiments, we found a most specific and a very high antagonistic activity of the endophyte *P. variabile* against common phytopathogens. In order to understand the mechanism involved in this phenomenon we characterized the main secondary metabolites produced by the endophytic fungus under standard growth conditions and assessed their activity on *F. oxysporum* growth. No inhibitory activity could be measured suggesting that the compounds involved in the antagonism are either produced in very small quantities or induced only at the time of the competition with the plant pathogen. In this context, a metabolomic study revealed the induction of diffusible metabolites by the endophyte when co-cultured with *F. oxysporum,* supporting the hypothesis of the specific production of defensive compounds by endophytic fungi during the microbial competitions. Extensive MS/MS analysis of the induced metabolites within the confrontation zone led to the characterization of two induced metabolites belonging to the oxylipin family. Moreover, the production of these oxylipins was associated with the decrease of the production of beauvericin, a common mycotoxin of *Fusarium oxysporum*. These results constitute the first experimental report supporting the hypothesis of induced defensive compounds in the course of a microbial competition between an endophytic fungus and a fungal phytopathogen and highlight the role of oxylipins in the phytopathogen-endophyte fungi interaction and in the oxylipin-mediated signalling in mycopathogens which is still in its infancy. Moreover, these results open a new research field on lipid-mediated signal communication with implication in quorum sensing and provide some perspective in the control of mycotoxigenic fungi by endophytic fungi in order to reduce the mycotoxin contamination in food and feed.

## Supporting Information

Figure S1 **CD spectrum of isosclerone (1).** Recorded in MeOH at 22°C (*c*, 10^−2^)
(TIF)

Figure S2 **Fragmentations spectrum of the positive ion** ***m*** **/** ***z*** **= 784.4.** This identified as beauvericin and some of the putative structures of the fragments are represented.
(TIF)
