# Supplementary figures and images for: Chemical Communication between the Endophytic Fungus Paraconiothyrium Variabile and the Phytopathogen Fusarium oxysporum
Source: PLoS One. 2012 Oct 15;7(10):e47313. doi: 10.1371/journal.pone.0047313 (PMC3471838; doi:10.1371/journal.pone.0047313)

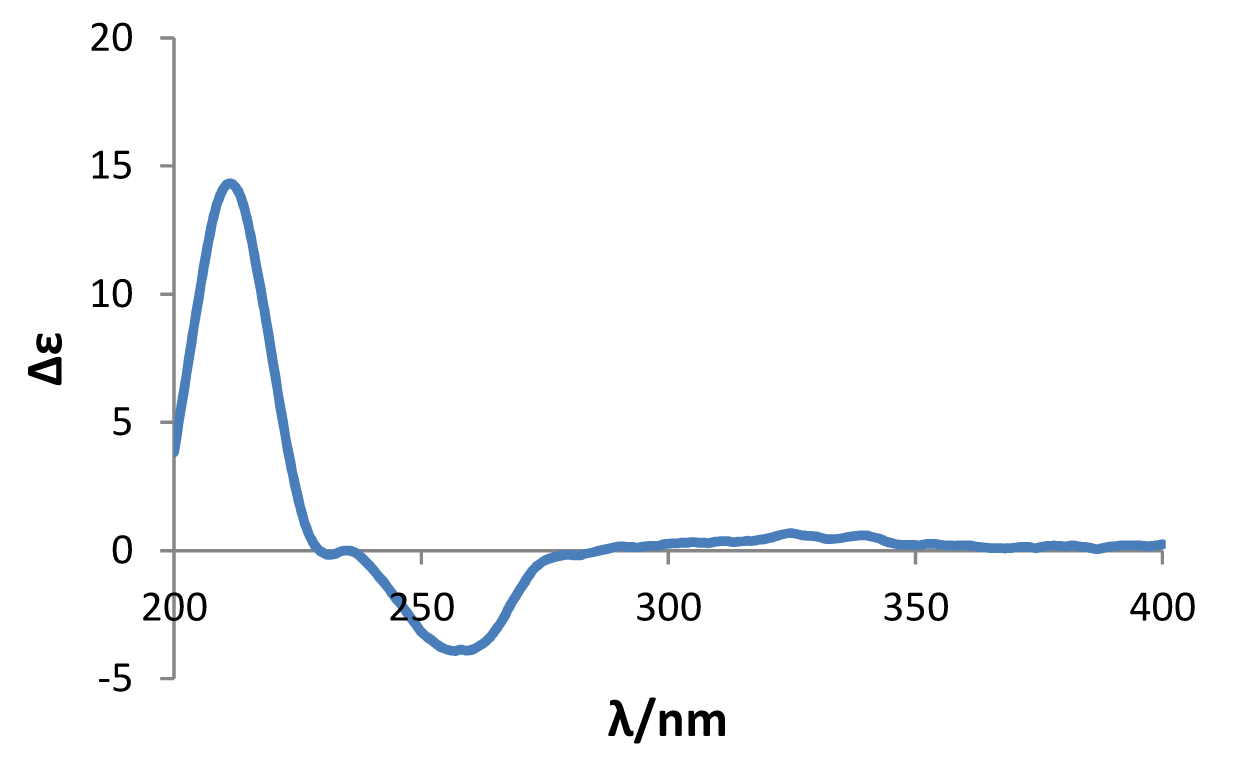

Supplement: Figure S1 — CD spectrum of isosclerone (1). Recorded in MeOH at 22°C (c, 10−2) (TIF) [file pone.0047313.s001.tif]

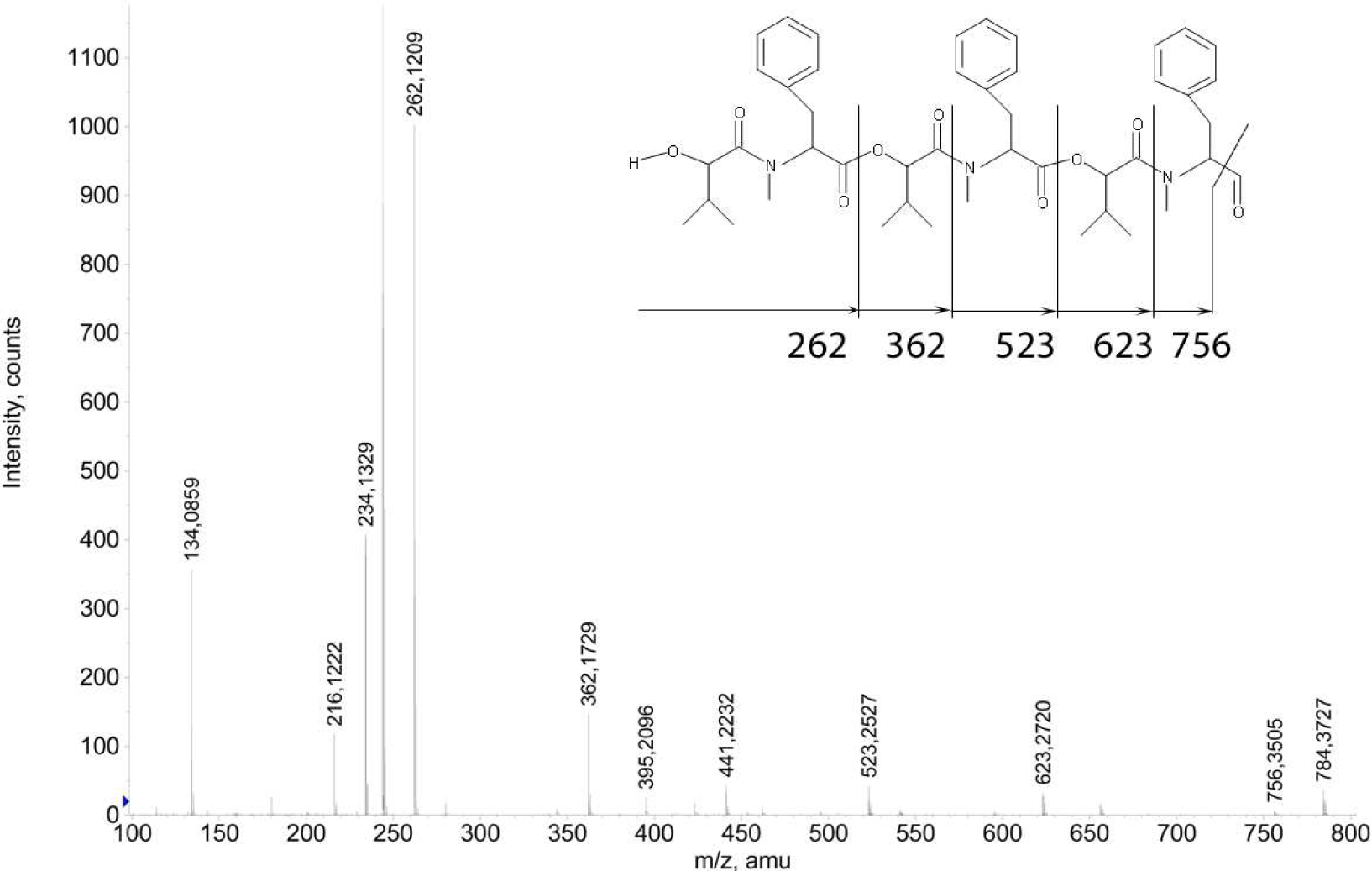

Supplement: Figure S2 — Fragmentations spectrum of the positive ion m / z = 784.4. This identified as beauvericin and some of the putative structures of the fragments are represented. (TIF) [file pone.0047313.s002.tif]
